# Chronic nonbacterial osteomyelitis in children: a retrospective multicenter study

**DOI:** 10.1186/s12969-015-0023-y

**Published:** 2015-06-19

**Authors:** Daniela Kaiser, Isabel Bolt, Michael Hofer, Christa Relly, Gerald Berthet, Dieter Bolz, Traudel Saurenmann

**Affiliations:** Department of Pediatric Rheumatology, Children’s Hospital, Kantonsspital Luzern, CH-6000 Luzern 16, Switzerland; Department of Pediatric Rheumatology, University Children’s Hospital, Berne, Switzerland; Department of Pediatric Rheumatology, CHUV, Lausanne and HUG, Geneva, Switzerland; Department of Pediatric Infectious Diseases, University Children’s Hospital Zurich, Zurich, Switzerland; Department of Pediatric Rheumatology, Children’s Hospital, Aarau, Switzerland; Department of Pediatric Rheumatology, University Children’s Hospital, Basel, Switzerland; Department of Pediatric Rheumatology, University Children’s Hospital, Zurich, Switzerland

**Keywords:** Nonbacterial osteitis, Chronic recurrent multifocal osteomyelitis, Sapho-Syndrome, Auto-inflammatory disease, Spondylarthritis

## Abstract

**Background:**

To determine the clinical presentation, current treatment and outcome of children with nonbacterial inflammatory bone disease.

**Methods:**

Retrospective multicenter study of patients entered into the Swiss Pediatric Rheumatology Working Group registry with a diagnosis of chronic nonbacterial osteomyelitis (CNO) and synovitis acne pustulosis hyperostosis osteitis (SAPHO) syndrome. The charts were reviewed for informations about disease presentation, treatment, course and outcome.

**Results:**

Forty-one children (31 girls and 10 boys) from 6 pediatric hospitals in Switzerland diagnosed between 1995 and 2010 were included in the study. The diagnosis was multifocal CNO (*n* = 33), unifocal CNO (*n* = 4) and SAPHO syndrome (*n* = 4). Mean age at onset of CNO was 9.5 years (range 1.4–15.6) and mean follow-up time was 52 months (range 6–156 months). Most patients (*n* = 27) had a chronic persistent disease course (>6 months), 8 patients had a course with one or more relapses and 6 patients had only one episode of CNO. Forty nine percent had received at least one course of antibiotics. In 57 % treatment with nonsteroidal anti-inflammatory drugs (NSAID) was sufficient to control the disease. Twelve out of 16 children with NSAID failure subsequently received corticosteroids, methotrexate, TNF α inhibitors, bisphosphonates or a combination of these drugs.

**Conclusions:**

In a multicenter cohort of 41 children 22 % started with unifocal lesion with a significant diagnostic delay. A higher proportion presented with chronic persistent disease than with a recurrent form. An osteomyelitis in the pelvic region is significantly associated with other features of juvenile spondylarthritis.

## Background

Chronic nonbacterial osteomyelitis (CNO) is a relatively rare pediatric rheumatic disease first described by Giedion *et al.* [1]. The 4 patients he reported suffered from “symmetrical” bone pain. Pain remains the cardinal feature of CNO. Symptoms of general disease as one would expect in acute infectious osteomyelitis were absent.

After this first description the diagnosis CNO was considered in children presenting with multifocal osteomyelitis [2, 3]. Observations of a greater diversity of the clinical presentation of CNO followed [4, 5]. Nowadays it is accepted that the presentation of aseptic osteomyelitis can be either unifocal [6, 7] or multifocal, acute (duration < 6 months) or chronic and the disease course is not always recurrent. Consequently, new terms such as nonbacterial osteitis (NBO) or chronic nonbacterial osteomyelitis (CNO) have been proposed [8, 9]. In some cases a multifocal disease is only apparent on diagnostic imaging as some bone lesions remain clinically asymptomatic.

This aseptic autoinflammatory condition of the musculoskeletal system affects preferentially children, sometimes adolescents. But osteitis is also part of the SAPHO syndrome which is more frequent in adults. 1987 Charmot *et al.* coined the acronym synovitis, acne, pustulosis, hyperostosis and osteitis (SAPHO) syndrome as a separate entity [10]. This syndrome is mainly associated with hyperostosis of the anterior chest wall and skin disorders of the type of neutrophilic dermatoses. These dermatoses are a group of inflammatory skin diseases of uncertain etiology [11] and include palmoplantar pustulosis (PPP), psoriasis, acne fulminans, neutrophilic eccrine hidradenitis, Sweet syndrome and pyoderma gangrenosum. In fact, CNO can be accompanied with neutrophilic dermatoses as aforementioned as well. This association, first described by Probst 1976 [12] can be seen in a sizeable proportion of cases and seems to be more common with increasing age of the patient [13, 14]. Therefore, it has been hypothesized that CNO may be the pediatric form of SAPHO syndrome [15]. Other authors have postulated that osteitis is the common component of a disease spectrum with different clinical presentations but the same etiology and pathophysiology [16].

Also an evolution of CNO towards spondylarthritis has been described in children and adults [17]. Spondylarthritis (SpA) in children is often undifferentiated at onset. The signs and symptoms at disease onset differ from those seen in adults, with inflammatory back pain being less common, reflecting the rare involvement of the sacroiliac and vertebral joints in juvenile disease. By contrast, hip and peripheral arthritis together with enthesitis are common presenting features in juvenile onset spondylarthritis [18]. In our study we compared a group of patients qualifying for juvenile spondylarthritis with the total cohort in order to evaluate whether these two groups can be distinguished early on. The next aim was to determine the features of nonbacterial osteitis in pediatric patients, the management, the course of the disease and the outcome.

## Patients and Methods

The Swiss Pediatric Rheumatology Working Group registry included all patients seen in the 6 pediatric rheumatology centers throughout Switzerland. The registry was searched for the diagnoses SAPHO syndrome and CRMO/CNO. In addition, other specialties such as pediatric infectious diseases, orthopedics or pediatric surgery at the same 6 centers were asked to contribute patients treated by them, if available. All medical records were reviewed, and data about history and clinical presentation, markers of inflammation and bone metabolism, HLA-B27, histological and radiological findings at presentation and during follow-up, medication used and outcome were collected using a standardized form and entered into an Excel spread sheet.

Based on the course of their disease patients were assigned to 3 different groups: 1. Patients with an acute form (single course less than 6 months duration); 2. Patients with a relapsing form (at least 2 flares with a symptom-free period in between without treatment); 3. Patients with a persistent form with complaints with or without treatment more than 6 months. Table 1

In addition, we divided the patients in one group with osteomyelitis +/− peripheral arthritis and another group with additional features of juvenile onset spondylarthritis such as axial arthritis, enthesitis, psoriasis and PPP, acute iridocyclitis, inflammatory bowel disease, HLA-B27 positivity or a family history of HLA-B27 associated disease (Table 2). Patients had to present at least one clinical feature (axial arthritis, enthesitis, psoriasis, PPP, acute uveitis or IBD) to be included into this group.

**Table 1:** Clinical and laboratory features of patients CNO

Patient	Sex (mm/ h)	Age	ESR	Course	Immun-suppressives	TNF	Bisphosphonates	Follow up
1	M	4.7y	32	Relapsing	No	No	No	5.8y
2	F	9.6y	12	Persistent	No	No	No	0.7y
3	M	1.4y	83	Persistent	MTX	No	Neridronat	8.7y
4	F	11.4y	16	Relapsing	No	No	No	3.8y
5	M	10.1y	38	Persistent	MTX	No	Neridronat	0.8y
6	F	8.3y	8	Persistent	MTX	No	No	2.5y
7	F	7.5y	46	Acute	No	No	No	
8	F	1.9y	30	Acute	No	No	No	15y
9	F	13.8y	25	Persistent	-	-	-	1.9y
10	M	9.5y	49	Relapsing	Steroids	No	No	7y
11	F	10.5y	54	Relapsing	Steroids	No	PAM	2.6y
12	F	4.1y	61	Relapsing	No	No	No	7.1y
13	F	8.8y	35	Relapsing	Steroids	Yes	PAM	10y
14	F	12.5y	7	Relapsing	No	Yes	No	4.8y
15	F	12.0y	60	Persistent	No	Yes	No	10.3y
16	F	12.5y	57	Acute	No	No	No	0.9y
17	F	9.0y	-	Persistent	Steroids	yes	PAM/Alendronat	5y
18	M	11.7y	56	Persistent	No	No	No	2y
*19*	*M*	*15.8y*	*18*	*Acute*	*No*	*No*	*No*	*6.3y*
20	F	11.4 y	5	Persistent	Steroids/MTX	No	No	1.5y
21	F	11.5y	19	Persistent	Yes	No	No	1.7y
22	F	9.9y	17	Persistent	No	No	No	7.5y
23	F	6.0y	6	Persistent	No	No	No	13y
*24*	*F*	*10.5y*	*34*	*Persistent*	*Steroids/MTX*	*No*	*No*	*8y*
25	F	9.9y	40	Acute	No	No	No	0.8y
26	F	11.5y	35	Persistent	No	Yes	No	2.4y
27	M	15.3y	8	Persistent	No	No	No	1.6y
28	F	10.5y	20	Persistent	No	No	No	4.2y
29	M	8.5y	55	Persistent	No	No	No	0.5y
30	F	9.2y	42	Persistent	No	No	No	3y
31	F	14.7y	18	Persistent	No	No	No	2.8y
32	F	8.9y	36	Persistent	No	No	No	4.8y
33	F	9.3y	52	Persistent	No	No	No	1.2y
34	F	7.5y	33	Acute	No	No	No	6.3y
*35*	*F*	*8.9y*	*43*	*Persistent*	*MTX*	*Yes*	*No*	*5.5y*
*36*	*F*	*1.8y*	*-*	*Persistent*	*MTX*	*No*	*No*	*1.y*
37	M	8.7y	31	Persistent	No	No	No	0.8y
38	F	6.1y	21	Persistent	Steroids/MTX	Yes	No	7.5y
39	F	9.5y	6	Persistent	No	No	No	1.3y
40	M	5.8y	53	Relapsing	Steroids/MTX	Yes	No	2.1y
41	F	10.3y	62	Persistent	Steroids/MTX	No	No	1.9y

In italics: Patients diagnosed with SAPHO (synovitis, acne, pustulosis, hyperostosis, osteitis) syndrome

Acute form: single course of <6 months duration. Relapsing form: at least 2 flares with remission in between. Persistent form with complaints with or without treatment for ≥6 months. NSAID sufficient to control pain

*PAM* pamidronat,*n.d*. not done, *PPP* palmoplantar Pustulosis

**Table 2:** Characteristics of patients with features of juvenile spondylarthritis

Patient	Gender	Age	HLA-B27	ANA	Derma	FH	Axial arthritis	Peripheral arthritis
8	F	1.9y	pos	pos	PPP	neg	No	Yes
9	F	13.5y	neg	n.d.	No	neg	Sacroileitis	No
14	F	12.5y	neg	neg	No	neg	Sacroileitis	Yes
15	F	12.0y	neg	n.d.	No	-	Sacroileitis	Yes
19	M	15.7y	n.d.	n.d.	No	-	Sacroileitis	No
24	F	10.1y	neg	neg	Psoriasis	-	Costo-vertebral	No
35	F	8.9y	pos	neg	Psoriasis	-	Sacroileitis	Yes
36	F	10.8y	pos	neg	PPP	pos	Yes	Yes
40	M	5.8y	n.d.	neg	PPP	No	No	No

*FH* Family History, *PPP* Palmoplantar Pustulosis

Mean, standard deviation and statistical significance (*T*-Test) was performed using Excel 2007 from Microsoft Corporation (Redmond, WA 98052 USA). The study was approved by the institutional ethics review boards of all participating centers.

## Results

### Patients’ characteristics and clinical presentation

**Fig. 1:**
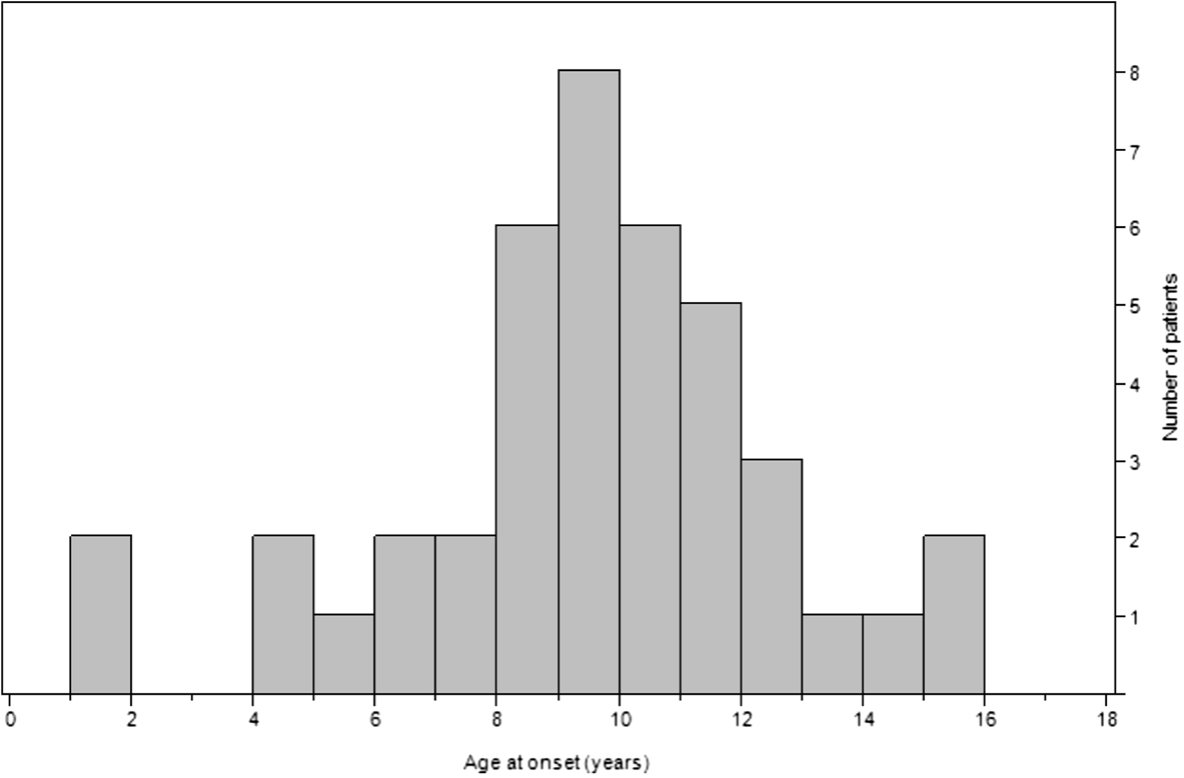
Distribution of patients by age at disease onset

We found 41 patients diagnosed between 1995 and 2010 with CNO (37 cases) or SAPHO syndrome (4 cases). Patients’ characteristics are shown in Table 2. Median age at onset was 9.5 ± 3.1 years (range 1.4–15 years) with a female predominance of 3:1. As shown in Fig. 1 most patients present between 7 and 12 years of age. The mean diagnostic delay was 8 months (range 1–64 months). Diagnostic delay in the group with unifocal disease was longer with 12.1 months versus 7 months in multifocal onset (*p* = 0.03). Patients were followed up for a median period of 52 months (range 0.5–14 years).

### Diagnosis

For all but 3 patients conventional X-rays were available for diagnosis. The most common additional diagnostic imaging method used was MRI (36 patients) for local lesions. Only in 5 / 41 patients a bone scintigraphy or CT was made instead of MRI. Four of the 41 patients initially had a normal radiograph of the symptomatic region. In these 4 patients the MRI showed typical findings of marrow edema with hypointense lesions on T1-weighted or hyperintense lesions on T2- weighted images and/or abnormal enhancement after intravenous administration of gadolinium-based contrast medium. In these 4 cases characteristic conventional radiographic signs such as osteolytic and/or sclerotic changes or periostal reactions were absent.

Bone biopsies were available for 29 out of 41 patients (71 %). Histological investigations showed nonspecific, inflammatory changes with granulocytic infiltration and fibrotic and/or hyperostotic regeneration.

At diagnosis, ESR was elevated (>15 mm/h) in 82 % (median 34 mm/h, range 5–83). Pathological laboratory test results such as ESR, CRP and thrombocytosis did not correlate with clinical signs of inflammation such as swelling, redness, local warmth or fever. HLA-B27 was positive in 5 / 24 patients tested (21 %). Two of these 5 patients had associated arthritis, psoriasis or palmoplantar pustulosis and had a diagnosis of SAPHO syndrome, both had sacroiliitis. Two other girls of these HLA-B27 positive patients had a vertebral osteitis.

**Fig. 2:**
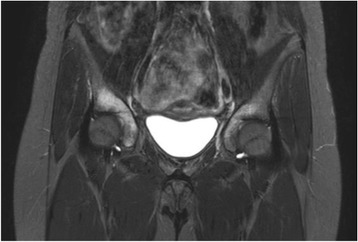
Case 1: Imaging data of CNO involving pelvis in a 12-year-old girl. Coronal STIR MRI reveal increased signal in the right and left acetabulum

Nine of the 41 patients had symptoms related to spondylarthropathy as sacroiliitis, costo-vertebral arthritis or psoriasis. There were no differences between this subgroup and the non-spondylarthropathy patients in regards to sex, age, ESR at onset, disease course and remission rate. The only significant difference was the occurrence of osteomyelitis in the pelvic region, which was present in all 9 patients of the spondylarthropathy subgroup but only in 1/32 patients of the other patients. This female patient with lesions at the acetabulum (Fig. 2) didn’t present a sacroiliitis or psoriatic skin lesion, but HLA-B27 was tested positive. Of the spondylarthritis group 6/9 patients had a radiological confirmed sacroiliitis, 5/9 had a psoriatic skin lesion. Overall 13 of 41 patients showed a spinal osteomyelitic lesion, thereof 5 at thoracic spine, 1 lumbar spine und 7 at the sacrum. Of these 13 patients with axial osteomyelitis only 6 patients had additional symptoms related to spondylarthropathy.

### Associated diseases

Skin lesions were present in 6 out of 41 children (17 %). Three patients presented with typical palmoplantar pustulosis. Psoriasis or acne was described in one patient each, 1 patient had both psoriasis and acne.

An associated autoimmune disease was seen in 2 out of 41 patients: 1 patient had a diagnosis of unclassified panniculitis and a girl had an additional diagnosis of ANA-positive juvenile idiopathic oligoarthritis with uveitis. No patient had inflammatory bowel disease.

### Treatment

Nineteen patients (46 %) initially received antibiotic treatment, 4 out of 9 patients with unifocal and 14 out of 32 with multifocal presentation. We found a statistically significant difference in the use of antibiotics across the centers (*p* = 0.02), but not according to disease characteristics such as unifocal presentation. Six patients also underwent surgery (2 bone resections, 4 bone curettages). Thirty-seven (90 %) received NSAIDs as anti-inflammatory drug. In 21/37 (57 %) this treatment was sufficient to control pain. Since radiologic follow up was not part of our study, the radiologic evolution of the bone lesions in these patients are not known. After failure of NSAIDs 12 children were treated either with systemic steroids (7 patients), methotrexate (7), TNF-blockers (7), anakinra (1) or bisphosphonates (5). Eleven out of these 12 patients (96 %) were treated with different drugs sequentially. Of the 7 patients treated with methotrexate, 6 received the drug for a period of 3–24 months without obvious improvement. Only 1 patient with SAPHO syndrome received methotrexate for 3.8 years, but the indication for MTX use in this case was peripheral arthritis. In our series no patient was treated with sulfasalazine.

Bisphosphonate was used in 5 patients with improvement in 2. One girl received a single cycle (3 infusions) of pamidronate and thereafter was in continuous remission until the last follow-up 20 months later. The other patient experienced relief of his bone pains with neridronate infusions 9 years after disease onset, but was not able to stop NSAID treatment. Only in 1 patient a combination of bisphosphonates with TNF-blocker was used. This girl was successfully treated during her fifth disease relapse with adalimumab and pamidronate.

Of the 8 patients treated with biologics only 2 were treated successfully for osteomyelitis with etanercept over a period of 12 and 28 months respectively. One patient had adalimumab for arthritis but had persistent osteomyelitis despite improvement of arthritis. In 2 cases treatment with TNF-blockers was stopped after a short time because of side effects (skin infection, allergic reaction) and in further 2 cases treatment was unsuccessful. One patient had anakinra without improvement.

In the 13 patients (32 %) with vertebral lesions the therapeutic approach and response to treatment was not different from the group without vertebral lesions.

### Disease course

At the onset of symptoms 9 patients (22 %) had a unifocal osteitis. Five of these 9 patients developed multifocal disease later on. The mean observation time in the unifocal course was 24 months in contrast to 72 months in the multifocal course of disease. Also, the only patient in our cohort with mandibular osteitis had only one disease focus. Six patients had an acute form and were symptom-free after 6 months during follow up. A non-recurrent, persistent form of illness was noted in 66 % (27 patients) whereas 8 patients (19 %) had a relapsing course. The patients with the recurrent course experienced between 1 and 5 relapses. The median time between the relapses was 24.5 months (range 7 to 90 months). Observation time in the group of the persistent form was 43.8 months; in the relapsing form 64.4 months. Nine children with chronic disease needed treatment for more than 5 years, some with NSAIDs alone, some with different drugs. The longest course of active illness was 13 years.

Complications included fractures at the site of inflammation in 2 patients. Both had a pathological fracture of a vertebral body at diagnosis. Another patient suffered from scoliosis due to wedging of a vertebral body, furthermore 1 patient had severe hyperostotic bone lesions and yet another one had bone length difference following surgical intervention.

## Discussion

In this retrospective study we report the disease characteristics of a group of 41 patients with chronic nonbacterial osteomyelitis collected from 6 Swiss pediatric centers. We were able to show the huge diversity of presentation, disease course and response to therapy.

### Diagnosis

Similar to other pediatric series, mean age at onset of nonbacterial osteomyelitis was 9.5 years with a predominance of females of 3:1 [19, 20]. CNO still remains a difficult diagnosis, so mean diagnostic delay in our cohort was 8 months. Possible reasons include the fact that plain radiographs are not sensitive enough to detect osteomyelitis or unifocal lesions were misdiagnosed as acute infectious osteomyelitis. This may also explain why the diagnostic delay in patients with a unifocal presentation was significant longer than in multifocal onset. Obviously histological examination is non-specific, but biopsy was helpful to exclude diseases like Langerhans cell Histiocytosis X, benign or malignant bone tumors especially in unifocal lesions [21].

Although today we assume that CNO belong to the family of autoinflammatory diseases with osseous manifestation, many patients with CNO and SAPHO-Syndrome have beside the osteitis, symptoms from the spectrum of the spondylarthritis as an axial involvement, the occurrence of IBD and an increased prevalence of the HLA-B27 phenotype [15]. This raises the question, whether CNO is a disease with different subgroups. Based on accompanying features we divided our patients into 2 subgroups, with and without features of juvenile spondylarthritis. In our cohort only 6 patients had arthritis, of which 5 had also sacroiliitis. Arthritis in CNO has been reported in up to 80 % in one serie [9], but most series report about 30 % (17, 23). Comparing the two subgroups we found no significant difference regarding disease presentation and course. But interestingly pelvic osteomyelitis was significantly associated with features of spondylarthritis, as all patients from the spondylarthritis group had an osteomyelitis in this localization but only one of the other 32 patients had. The pelvis is a typical site of CNO with 11–34 % of patients affected [8, 22–24], which is consistent with our findings (25 %). This is a fact which may be helpful in the future to distinguish subgroups of patients with CNO. On the other hand we couldn’t find a difference in spinal involvement comparing the two groups. Hence in our study-population an axial lesion is not a criteria for the evolution of spondylarthritis, as it can be seen in CNO. The frequency of HLA-B27 is low in CNO compared to patients with ERA, 21 % in our population.

### Treatment

Pathogens such as proprionebacterium acnes are no longer considered relevant in the pathogenesis of CNO, as today CNO is placed in the category of autoinflammatory diseases. Nevertheless, half of our patients were treated with antibiotics first, but with a significant difference across the centers. There was no difference between the use of antibiotics for unifocal or multifocal presentation.

Fifty-seven percent of our patients responded well to the treatment with nonsteroidal anti-inflammatory drugs (NSAID), which is in keeping with data published in other reports [25, 26]. NSAID can control pain, which doesn’t mean there is remission radiologically [26]. In case pain does not respond to NSAIDs, a short course of corticosteroids may be an alternative. Methotrexat (MTX) is an approved drug in children with rheumatologic disorders, also several cases of SAPHO-syndrome responsive to methotrexat therapy have been reported [15]. Nevertheless, MTX therapy was ceased in all but one of our patients because of lack of improvement. In a cohort of 70 children with CNO reported by Borzutzky *et al.* [24] 20 % had clinical remission treated with methotrexate. They observed the highest rate of clinical remission with TNF-α inhibitors (46 %). Several other case reports describe the efficacy of anti-TNFα therapy [27, 28]. In our population we found mixed success with TNFα-agonists as well as with bisphosphonate therapy.

The good effect of bisphosphonate therapy has been documented in several reports [29–31]. Rodrick *et al.* found a good or moderately good response in 8 out of 11 patients (73 %) to pamidronate therapy, bone lesions resolved or showed significant improvement in the second WB-MRI. The improvement of bone inflammation after pamidronate therapy was also reported by Hofmann *et al.* [32]. Although no complete radiological remission could be achieved, bisphosphonates are an optional treatment for patients with vertebral lesion to prevent fractures and orthopedic complications as shown by Hospach *et al.* [33], while fractures occurred often (up to 40 %) in vertebral involvement [34]. However in the face of the long half-life time of bisphosphonates and the side effects, the indication for bisphosphonate therapy has to be made carefully.

The inclusion of patients with this rare disease in a large registry (for example www.printo.it/eurofever) as initiated by the Paediatric Rheumatology International Trials Organisation PRINTO will be helpful to determine an effective treatment.

### Disease course

In contrast to the previous assumption that nonbacterial osteomyelitis is a recurrent disease, the majority of our patients (67.5 %) suffered from a chronic persistent illness. This is a higher proportion than described in other cohorts. Gikas at al found 49 % with a non-recurrent disease pattern [22].

We saw that disease activity may persist for years or even decades. In our cohort 9 children (22 %) with chronic disease needed treatment for more than 5 years. The longest course of active illness was 13 years. Only 30 % came into remission, which is similar to the findings of Catalano-Pons *et al.* [19] in an equally large cohort. In their study 58.6 % of patients had active disease at follow up (0.5–15 years after diagnosis). More than 25 % of the cohort examined by Huber *et al.* [23] had persistent CNO activity at the time of evaluation a median of 12 years later and after a median overall duration of active disease of 5.7 years. In the follow-up study of Duffy *et al.* [35] the duration of symptoms ranged from 2.5 to 20 years. Huber *et al.* [30] conclude that CRMO usually has a favorable evolution with no major sequelae. This is in keeping with our findings, where also only 4 patients had orthopedic complications during the observation period of 52 months (range 6 months to 14 years).

Vittecoq *et al.* concluded [17] that CRMO usually evolved to spondylarthropathy. Despite special considerations of these features we can’t agree with this evolution in our cohort, because 32 of our 41 patients still had osteomyelitis at the end of the observation period. As described by Zibroswska-Bech *et al.* [36] we found the extra-osseous manifestation typically present at the time of diagnosis.

Our study is limited by the retrospective multicenter and multidisciplinary design, which does not allow for analyses regarding disease details and treatment. However, despite these shortcomings we think our findings may still be helpful to improve knowledge and enhance awareness about this unique disease across the involved disciplines.
